# Therapeutic Role of Mobilized Bone Marrow Cells in Children with Nonischemic Dilated Cardiomyopathy

**DOI:** 10.5402/2012/927968

**Published:** 2012-10-21

**Authors:** Nevin M. Habeeb, Omneya I. Youssef, Eman S. El Hadidi

**Affiliations:** Pediatrics and Clinical Pathology Departments, Faculty of Medicine, Ain Shams University, Cairo 11321, Egypt

## Abstract

Dilated cardiomyopathy is an important cause of congestive cardiac failure in infants and children. Mobilizing hematopoietic progenitor cells is a promising intervention to this deadly disease. *Aim*. Evaluate granulocyte colony stimulating factor (GCSF) as therapeutic modality in children with idiopathic dilated cardiomyopathy (IDCM). *Subjects and Methods*. This case-control prospective study was conducted on 20 children with IDCM following up at Cardiology Clinic Children's Hospital, Ain Shams University (group 1) who were compared to another 10 age-, sex-, duration-of-illness-, and systolic-function-matched children with IDCM as control (group 2). They were subjected to history taking, clinical examination, echocardiography, and peripheral blood CD34+ cell assessment before and one week after GCSF intake for 5 consecutive days (by group 1 but not group 2). *Results*. A significant improvement in echocardiographic data and CD34+-T-cell increase was found in group 1 one week after GCSF intake and for the next 6 months CD34+ T cells percentage of change showed no significant correlation with the that of the left ventricular dimensions and systolic function. *Conclusion*. Administration of GCSF to children with IDCM resulted in clinical and echocardiographic improvement not correlated to mobilized CD34+ T cells, implying involvement of additional mechanisms over simple stem cell mobilization.

## 1. Introduction

Dilated cardiomyopathy is an important cause of chronic congestive cardiac failure in infants and children. Although a variety of etiological factors have been listed, most patients with echocardiographically documented dilated cardiomyopathy do not possess a demonstrable cause [1].

Poor myocardial function in dilated cardiomyopathy triggers a sequence of compensatory mechanisms that favor myocardial and peripheral vascular remodeling by necrosis, fibrosis, and apoptosis which ultimately do more harm than good [2].

Medical intervention will remain the cornerstone of management until advances in surgical techniques become more widely available [3].

Mobilizing hematopoietic progenitor cells to repair the failing heart is a promising intervention to halt the progression of this deadly disease. Low doses of GCSF, five microgram per kilogram per day, were found to improve systolic function in adults with advanced systolic heart failure [2].

Bone marrow stem cells were found to contribute to the regeneration of nonhaematopoietic organs. Data from preclinical models indicate that cluster of differentiation thirty-four cells restore the microcirculation and improve myocardial tissue perfusion [4]. Recent studies have shown that granulocyte colony stimulation factor may enhance bone marrow cell migration to damaged heart in increased apoptosis and Fas protein expression [3].

## 2. Subjects and Methods 

This case-control prospective study was conducted on twenty children with idiopathic dilated cardiomyopathy following up at cardiology clinic Children's Hospital, Ain Shams University (group 1).

**Inclusion Criteria**

Ejection fraction less than forty-five percent and left ventricular dilatation [5], patients diagnosed with IDCM for one year or longer, and kept on antifailure medications for at least 6 months prior to the study with no improvement of echocardiographic parameters.

**Exclusion Criteria**

Patients with genetic syndromes, active myocarditis, and systemic, genetic, endocrinal, and metabolic diseases causing cardiomyopathy.

Patients were compared to ten age- and sex-, duration-of-illness-, and systolic-function-matched children with ICDM as a control group (group 2). An informed consent was taken from parents of studied children or care givers.

The study was carried out over three phases.

**Phase One: Before Treatment**

Full history taking, laying stress on cardiac symptoms/heart failure symptoms as dyspnea and orthopnea, to grade the patients according to the New York Heart Association classification criteria.

- Thorough clinical examination stressing on signs of heart failure.
- Twelve lead electrocardiography to diagnose any associated rhythm abnormalities.
- Motion mode, two dimensional, color-pulsed, and continuous wave doppler echocardiography for assessment of cardiac chamber size, valve regurgitation, and systolic as well as diastolic function.
- Complete blood count was done by coulter to ensure that the white blood cell, hemoglobin, platelet, and blood cell morphology were within normal limits.

Assessment of CD34+ T cells in peripheral blood was done using flow cytometry; two milliliters fresh peripheral venous blood samples were collected from patients on potassium ethylene diamine tetraacetate in a final concentration of one point five milligrams per milliliters.

Peripheral blood samples were stained with phycoerythrin conjugated monoclonal antibodies to CD34+ or isotypic control (Beckman Coulter, USA). Five microliters of each monoclonal antibodies were added to fifty microliters of anticoagulated blood and incubated for twenty minutes in dark. The cells were washed using phosphate buffer saline (Oxoid, England) and lysed using one milliliter lysing reagent (Beckman Coulter, USA). 

After appropriate gating, surface cluster of differentiation thirty-four expression was determined. Data acquisition and analysis were performed on EPICS XL flow cytometer (Beckman Coulter, USA) using system two version three software with a standard three-color filter configuration [6].

## 3. Interpretation

Positivity was considered when at least ten percent of the cells express cluster of differentiation thirty-four.

Group 1 patients were given GCSF, five micrograms per kilogram per day via subcutaneous route for five doses, for five consecutive days.

No concomitant changes were done to antifaliure medication types or doses throughout the study.

**Phase Two: After Group 1 GCSF Treatment**

Patients of group 1 and group 2 were assessed clinically to obtain the New York Heart Association class and by echocardiography seven days from starting the first CGSF dose in group 1.

Assessment of CD34+ T cells in peripheral blood was done on both groups using flow cytometry to document haemopoietic cell mobilization.

No concomitant changes were done to antifaliure medications received by the patients six month before and throught the entire study.

## 4. Statistical Analysis 

Statistical analysis was performed using Statistical Package for Social Sciences, Version fifteen (Chicago, USA) for Windows. Continuous variables were analyzed as mean values plus or minus standard deviation. Rates and proportions were calculated for categorical data.

Kolmogorov-Smirnov test of normality was done to assess normality of continuous variables before starting the analysis. Paired *t* test was used to compare results before and after treatment (paired data). Differences among continuous variables with normal Student's *t* test and its nonparametric analogue Mann Whitney test was used for not normally distributed ones. McNemar-Bowker Test was used to compare categorical variables before and after treatment. All tests were two tailed, *P* values less than 0.05 were considered significant and less than 0.001 were considered as highly significant.

## 5. Results

This is a prospective case-control study conducted on 20 patients with IDCM (group 1). They were 12 males and 8 females. The mean and the standard deviation of their ages was 6.8 and 5.2 years, respectively. Ten healthy age- and sex-, duration-of-illness-, and systolic-function-matched children with IDCM served as a control group (group 2).

### 5.1. Results of Phase One

Clinical assessment revealed that 10% of patients of group 1 (2 patients) and group 2 (1 patient) patients were in NYHA class one, 60% of group 1 patients (12 patients) and group 2 patients (6 patients) were in NYHA class two while 30% of group 1 patients (6 patients) and group 2 patients (3 patients) were in NYHA class three.

Echocardiographic data of patients before group 1 treatment is presented in Table 1.

Serum troponin I level was elevated in five patients of group 1 (0.6 ng/mL in 2 patients, 0.4 ng/mL in 2 patients, and 0.3 ng/mL in one patient) and 4 patients of group 2 (0.6 ng/mL, 0.6 ng/mL, 0.4 ng/mL, and 0.3 ng/mL).

Cluster of differentiation 34 T cells of group 1 patients before treatment showed a mean of 0.04 and standard deviation of 0.03. The mean of CD34+ T cells of group 2 was 0.02 and the standard deviation was 0.04.

### 5.2. Results of Phase Two

There was significant improvement of the New York Heart Association class of group 1 patients (Table 2).

There was significant improvement of the echocardiographic data of group 1 patients, as well as significant increase of their CD34+ T cells (Table 1). 

Serum troponin I levels decreased below detectable range (below 0.2 ng/mL) in group 1 patients who showed elevated values in phase one except for one patients in whom troponin I values dropped from 0.6 to 0.3 ng/mL after Granulocyte Colony-Stimulating Factor intake. Serum troponin level slightly dropped but remained elevated in the 4 patients of group 2 (0.3 ng/mL, 0.4 ng/mL, 0.3 ng/mL, and 0.3 ng/mL).

There was significant correlation between the parameters of left ventricular systolic function (ejection fraction and fractional shortening) and the left ventricular end diastolic diameter in group 1 after treatment.

The percentage of change of the cluster of differentiation thirty-four T cells showed nonsignificant correlation with the percentage of change of the left ventricular dimension and systolic function (Table 3).

## 6. Discussion

Dilated cardiomyopathy is an important cause of chronic congestive heart failure in infants and children [1]. Despite various causes of dilated cardiomyopathy, the pathological feature of the disease is characterized by less functioning cardiomyocytes by which the myocardium fails to maintain normal contractile function [7].

Forty percent of children with symptomatic dilated cardiomyopathy are resistant to medical treatment [8]. Alternative therapeutic options have to be considered for dilated cardiomyopathy children with advanced heart failure [9].

Until recently it was thought that only embryonic stem cells are pluripotent; however, this concept has been changed and it has been shown that adult stem cells also possess plasticity [10].

Cellular cardiomyoplasty is considered as a new and promising therapeutic option for cardiac repair. Numerous studies in adult ischemic heart disease have been reported in both human patients and animal models [11]. According to the results of these studies, the autologous bone marrow cells transplanted into the ventricular scar tissue may differentiate into cardiomyocytes and improve cardiac function. In clinical trials mononuclear bone marrow derived cells have been intensively investigated. Clinical feasibility, safety, and short term outcomes are encouraging [12].

Mobilization of bone marrow cells into peripheral blood by certain cytokines such as granulocyte colony-stimulating factor offers a noninvasive therapeutic strategy for regeneration of myocardium after myocardial infarction [13]. However, there are few reports of experimental and clinical studies about the cell mobilization in idiopathic dilated cardiomyopathy in children [9].

In the current study, upon treating our recruited patients (group 1) with GCSF they showed marked clinical improvement in the form of regression of their New York Heart Association classification 7 days from the onset of GCSF treatment and for six months after. This improvement was also objectively documented by echocardiography which showed increase of ejection fraction, fractional shortening, and decrease of left ventricular dimension. Concomitantly there was significant rise of cluster of differentiation thirty-four T cell. Clinical and echocardiographic improvement were not shown in ICDM patients who did not receive GCSF (group 2).

 Hüttmann and coworkers, 2006 showed that GCSF administration improved physical performance not only in patients with ischemic cardiomyopathy but also in those with dilated cardiomyopathy [14].

Kakihana and coworkers, 2009, reported a case of nonischemic dilated cardiomyopathy in a patient with thromboangitis obliterans in whom cardiac function improved after GCSF mobilized peripheral blood mononuclear cells implantation on his ischemic leg. Their report suggested that peripheral blood mononuclear implantation with GCSF could be an effective approach to treating nonischemic heart failure, though the exact mechanisms of improved cardiac function are still unclear [15].

Harada and coworkers, 2005 and Dimmeler and coworkers, 2008, reported that GCSF promotes survival of cardiac myocytes and prevent left ventricular remodeling after myocardial infarction [16, 17].

There are several possibilities concerning the changes in cardiac function after GCSF mobilized cells. Zohlnhöfer and coworkers, 2008 stated that GCSF mobilizes stem cells or progenitor cells from bone marrow into injured myocardium and accelerates endothelial regeneration [13]. GCSF also protects cardiomyocytes and endothelial cells from apoptotic cell death [16]. GCSF has been reported to prevent left ventricular remodeling and dysfunction after myocardial infarction [13].

The current study showed no significant correlation between the percentage of rise of CD34+ T cells and the percentage of increase of the ejection fraction and fractional shortening of patients. This result suggests that there is another mechanism by which the GCSF improves myocardial function in children with IDCM dilated beside the effect of the mobilized bone marrow cells. Hüttmann and coworkers, 2006 suggested a direct action on the cardiac adrenergic nervous system which may be involved in the effect of GCSF [14].

The underlying mechanism of how mobilized CD34+ contribute to improvement of cardiac remodeling in pediatric dilated cardiomyopathy patients remains to be elucidated. The best scenario of mobilized cell fate is that cells differentiate into functioning cardiomyocytes within a failing heart, replacing damaged cardiomyocytes. However, studies have shown that rate of cardiomyocyte differentiation from stem cells is low and the mobilized cells are involved in angiogenesis and host cardiomyocyte regeneration via direct cell differentiation and/or paracrine effects that secrete various growth factors and/or cytokines [15].

Cellular cardiomyoplasty for pediatric dilated cardiomyopathy patients can be a new and promising therapeutic option that significantly reduces heart transplantation cases. This study provides new insights into investigation of a new therapy for pediatric dilated cardiomyopathy patients with advanced heart failure. Further clinical and basic researches are necessary.

## 7. Conclusion

Administration of GCSF to children with idiopathic dilated cardiomyopathy can result in clinical and echocardiographic improvement. This improvement was not correlated to the degree of stem cell mobilization.

To our knowledge this is the first study conducted on such a number of children with idiopathic dilated cardiomyopathy. We recommend more studies on larger number of patients and follow-up studies to patients who improved for evaluation of duration and progression of such improvement.

## Figures and Tables

**Table 1:** Comparison between echocardiographic data and cluster of differentiation 34 T cells of group 1 patients before and after treatment.

Echo parameter	Before treatment		After treatment		Percentage of change	Test value	*P* value
	Mean	Standard deviation	Mean	Standard deviation			
Ejection fraction	31.2	9.2	46.9	7.9	50.3	8.472	<0.001
Fractional shortening	15.0	4.8	23.2	4.3	54.9	7.752	<0.001
Left ventricular end diastolic diameter (cm)	4.8	1.0	4.6	0.9	−4.2	1.751	0.096
Cluster of differentiation 34 T cells	0.04	0.03	0.13	0.09	200	5.174	<0.001

**Table 2:** Comparison between New York Heart Association class of group 1 patients before and after GCSF treatment.

	New York Heart Association classification before treatment			
After treatment	I	II	III-IV	*P* value
	Number = 2 (10%)	Number = 12 (60%)	Number = 6 (30%)	
I	2 (100)	11 (91.7)	2 (33.3)	
II	0	1 (8.3)	2 (33.3)	0.002
III-IV	0	0	2 (33.3)	

**Table 3:** Correlation between the percentage of change of the echocardiographic data and the cluster of differentiation 34 T cells in group 1 after treatment.

Studied echocardiographic and laboratory parameters		Ejection fraction percentage of change	Fractional shortening percentage of change	Left ventricular end diastolic diameter percentage of change	Cluster of differentiation 34 T cells percentage of change
Ejection fraction percentage of change	*r*	1			
	*P* value	—			
Fractional shortening percentage of change	*r*	.986 (^*∗∗*^)	1		
	*P* value	<0.001	—		
Left ventricular end diastolic diameter percentage of change	*r*	−.149	−.085	1	
	*P* value	0.530	0.722	—	
Cluster of differentiation 34 T cells percentage of change	*r*	.119	.087	.116	1
	*P* value	0.616	0.714	.625	—
